# IgG-class anti-PF4/heparin antibodies and symptomatic DVT in orthopedic surgery patients receiving different anti-thromboembolic prophylaxis therapeutics

**DOI:** 10.1186/1471-2474-12-22

**Published:** 2011-01-24

**Authors:** Satoru Motokawa, Takafumi Torigoshi, Yumi Maeda, Kazushige Maeda, Yuka Jiuchi, Takayuki Yamaguchi, Shinsuke Someya, Hiroyuki Shindo, Kiyoshi Migita

**Affiliations:** 1 Department of Orthopedics Surgery and Department of Rheumatology, NHO Nagasaki Medical Center, Japan; 2 Clinical Research Center, NHO Nagasaki Medical Center, Kubara 2-1001-1, Omura 856-8652, Japan; 3 Department of Orthopedics Surgery, Nagasaki University School of Medicine, Sakamoto 1-7-1, Nagasaki 852-8501, Japan

## Abstract

**Background:**

Heparin-induced thrombocytopenia (HIT) is a thromboembolic complication that can occur with unfractionated heparin (UFH) or low molecular weight heparin (LMWH). Our objective was to determine and compare the incidence of IgG-class HIT antibodies in patients undergoing total hip arthroplasty (THA) or total knee arthroplasty (TKA) with different antithrombotic prophylaxis therapies and their contributions to the occurrence of venous thromboembolism (VTE).

**Methods:**

A prospective observational study was performed for 374 Japanese patients undergoing THA or TKA to determine the incidence of VTE. IgG-class anti-PF4/heparin antibodies were measured using IgG-specific EIA before and after the operation.

**Results:**

In the clinical outcome, the incidence of symptomatic deep vein thrombosis (DVT) was 15.0% (56/374, TKA; 35, THA; 21) and pulmonary emboli (PE) were not observed. The total seroconversion incidence of IgG-class PF4/heparin antibodies was 19.8% (74/374). The seroconversion incidence of IgG-class PF4/heparin antibodies was higher in patients receiving UFH (32.7%) compared to those receiving LMWH (9.5%) or fondaparinux (14.8%). Furthermore, the seroconversion incidence was significantly higher in patients undergoing TKA compared to those undergoing THA. Based on multivariate analysis, seroconversion of the IgG-class PF4/heparin antibodies was independent a risk factor for symptomatic DVT.

**Conclusion:**

Our findings show that the seroconversion of IgG-class anti-PF4/heparin antibodies differed with various anti-thrombotic prophylaxis therapeutics and was associated with the risk of DVT in a subset of patients undergoing total joint arthroplasty (TKA and THA).

## Background

Venous thromboembolism (VTE) is a serious complication of major orthopedic surgery including total hip arthroplasty (THA) and total knee arthroplasty (TKA) [1]. The incidence of postoperative deep vein thrombosis (DVT) is 45-57% after THA and 41-85% after TKA if prophylaxis is not used [2]. Consequently, pharmacological thromboprophylaxis is recommended and widely used in patients undergoing orthopedic surgery [3]. Although, low-dose unfractionated heparin (UFH) has been used as a thromboprophylactic agent, enoxaparin and fondaparinux have recently been approved for thromboprophylaxis in patients after TKA or THA in Japan [4,5]. LMWHs is an important tool in DVT management, offering advantages over UFH, considering the reduced risk for HIT [6], a prothrombotic adverse drug reaction caused by platelet-activating antibodies that recognize the complex of platelet factor 4 (PF4) bound to heparin [7]. Indeed, HIT is found in approximately 5% of patients receiving unfractionated heparin thromboprophylaxis in orthopedic surgery studies [8]. By contrast, a reduced risk of HIT has been confirmed in patients receiving LMWH or fondaparinux [9]. HIT is caused by the generation of heparin-dependent antibodies against the PF4/heparin complex, which cause platelet activation and aggregation and eventually progress to thrombocytopenia and thrombosis [10]. Recent studies have confirmed a minor role of IgM- and IgA- class anti-PF4/heparin antibodies in HIT [11,12] and there is growing evidence that only antibodies of the IgG class are capable of inducing platelet activation [13]. The aim of the present study was to determine the risk factors of VTE, and the incidence of symptomatic DVTs and PEs in patients undergoing THA or TKA under pharmacologic prophylaxis. Furthermore, in this study, we used a specific immunoassay that detects only IgG-class anti-PF4/heparin antibodies to determine the frequency of these antibodies and their contributions to the occurrence of VTEs.

## Methods

### Patients

All patients who underwent THA or TKA at our institution between September 1 2006, and April 30 2010, were enrolled in this study to determine the incidence of PE and symptomatic DVT. Data were collected regarding baseline patient characteristics, including age, gender, underlying disease and VTE risk factors (previous thrombosis, malignancy, diabetes, hypertension, hyperlipidemia and arrhythmia). Bleeding was defined as overt if it was clinically evident and there was a clear source of bleeding. None of the patients had any history of previous heparin exposure within the past 90 days. The study protocol was approved by the Ethics Committees of the Nagasaki Medical Center and written informed consent was obtained from each patient.

### Thromboprophylaxis Regimen

Our institution has used unfractionated heparin (UFH) since 2004. Patients received 1000 units of UFH via a single bolus intravenous injection during the operation and 5000 units of UFH via drip intravenous infusion (24 hr) on post-operative day 2. Since June 2007, fondaparinux and enoxaparin have been approved for post-operative thromboprophylaxis in Japan. Four months later, we changed the thromboprophylactic regimen to fondaparinux or enoxaparin in our institute. Fondaparinux (2.5 mg once a day) or enoxaparin (2,000 IU twice a day) were injected subcutaneously for 10 days. The operation was performed under general anesthesia in all cases. In TKA, air tourniquent was used during operation in all cases. All TKAs were performed with a cemented component. THAs were almost performed with a cementless femoral stem and an acetabular component. A foot pump (A-V Impulse System, Novamedix Corp, Hampshire, UK) and compression stockings were initiated on day 1 in all subjects.

### Detection of DVT/PE

After the operation, primary screening for VTE was undertaken by meticulous observation of the clinical signs, and determination of plasma D-dimer levels. For the clinical signs of DVT-PE, we carefully examined the patient for swelling of the entire leg or localized leg swelling, acute cardiovascular dysfunction, dyspnea and chest pain. Patients who exhibited high plasma concentrations of D-dimer >10 μg/ml, or were suspected to exhibit the clinical signs of DVT were subjected to CT scanning using MDCT from the chest to the ankle and venous Doppler ultrasonography for detection of DVT/PE.

### Blood sampling

Serum samples were collected before the operation and at postoperative day 7, and stored at -70°C. A sandwich ELISA kit (GTI Diagnostics, Waukesha, WI) was used to measure the IgG-class anti-heparin-PE4 antibody (HIT Ab) titer of the serum according to the manufacturer's instructions. The microtiter wells were precoated with polyvinylsulfonate-PF4 complexes. Antibodies bound to polyvinylsulfonate-PF4 were identified by affinity-purified anti-human IgG peroxidase conjugate. ELISA reactivities (optical density, OD) are expressed relative to a standard control. Positive and negative controls were included in every plate. The samples with readings ≥0.40 absorbance units were considered positive according to the manufacturer's instructions. While the results of the ELISA test merely confirmed the presence of HIT antibodies, HIT was defined as a >50% reduction in the platelet count or an absolute platelet count <10^5^/μl during and after heparin treatment, with no other cause of thrombocytopenia, and a positive result in the HIT antibody test [14].

### Assessment of bleeding

During this study, the patients were examined daily for any evidence of wound hematoma, as well as other manifestations of bleeding. Bleeding was considered to be major if there was a wound hematoma necessitating surgical revision, gastrointestinal bleeding, or any bleeding requiring transfusion of more than 400 ml of blood and interruption of prophylaxis.

### Statistical Analysis

Crude associations for categorical variables were evaluated using a Chi-squared test. For continuous variables, differences between groups were evaluated by Mann-Whitney *U *test. Multivariate analysis was conducted by fitting logistic regression analysis models to adjust for the effects of confounding factors while identifying significant predictors of DVT. The variables on multiple logistic regression analysis were selected from variables showing a *p *value <0.1 in univariate analyses. The exact confidence intervals and probability values are reported for the multivariate models. For all other tests, a *p *value <0.05 was considered statistically significant. All statistical analysis was performed with SPSS version 13.0 (Chicago, IL, USA). Odds ratios (OR) for regression modeling and their 95% confidence intervals (CI) are reported.

## Result

### Clinical outcome

#### Occurrence of VTE

The total joint arthroplasty (TKA and THA) were performed and the distributions of the patient's mean age and gender are presented in Table 1. Two hundred and fourteen patients (57.2%) underwent THA and 160 patients (42.8%) underwent TKA. For pharmacological VTE prophylaxis, unfractionated heparin (UFH), low molecular-weight heparin (LMWH; enoxaparin) and fondaparinux were administered in 27.8%, 25.4% and 21.7% of the total patients respectively. The primary diagnosis was osteoarthritis in 89.7% and 80.6% of the patients who underwent THA and TKA, respectively. Pulmonary emboli did not occur in this study. As shown in Table 2, symptomatic DVT was observed in 21 patients undergoing THA (9.8%) and 35 patients undergoing TKA (21.9%). All of the DVTs were the distal type.

**Table 1:** Patients characteristics

Characteristics	THA		TKA		*p*
	n = 214		n = 160		
Age	65.6 ± 11.7	(25-90)	73.9 ± 8.9	(30-89)	<0.00001
Age≧75 yr	49		89		<0.00001
Age < 75 yr	165		71		
Gender(male/female)	34/180		31/129		0.379
BMI(Kg/m^2^)	24.1 ± 3.8	(15.1-37.7)	25.9 ± 4.3	(13.7-41.4)	<0.00001
BMI≧30	17		30		0.002
BMI < 30	197		130		
					
Risk factors	102/214	(47.7%)	112/160	(70.0%)	<0.00001
OA/RA	192/22		129/31		0.013
Seroconvertion of IgG-class Anti-PF4/heparinAb	30/214	(14.0%)	44/160	(27.5%)	0.001
Treatment					
UFH	60/214	(28.0%)	44/160	(27.5%)	0.909
LMWH	64/214	(29.9%)	31/160	(19.4%)	0.021
Fondaparinux	49/214	(22.9%)	32/160	(20.0%)	0.501
Others	21/214	(9.8%)	29/160	(18.1%)	0.019
Aspirin	16/214	(7.5%)	27/160	(16.9%)	
Warfarin	4/214	(1.9%)	1/160	(0.6%)	
Cilostazol	14/214	(6.5%)	23/160	(14.4%)	
No medication	20/214	(9.3%)	24/160	(15.0%)	0.093

Abbreviations: BMI; body mass index, TKA; total knee arthroplasty, THA; total hip arthroplasty, OA; osteoarthritis, RA; rheumatoid arthritis, UFH; unfractionated heparin, LMWH; low-molecular weight heparin

**Table 2:** Incidence of DVT and major bleeding

THA/TKA	drug	No. patients	No. DVT(%)	No. bleeding(%)
**THA**	UFH	60	6(10.0)	3(5.0)
	LMWH	64	6(9.4)	1(1.6)
	Fondaparinux	49	4(8.2)	3(6.1)
	others	41	5(12.2)	1(2.4)

**TKA**	UFH	44	10(22.7)	5(11.4)
	LMWH	31	8(25.8)	2(6.5)
	Fondaparinux	32	4(12.5)	0(0)
	others	53	13(24.5)	5(9.4)

Abbreviations: DVT; deep vein thrombosis, TKA; total knee arthroplasty, THA; total hip arthroplasty, UFH; unfractionated heparin, LMWH; low-molecular weight heparin

#### Occurrence of bleeding

Twenty (5.3%) of the 374 operations were associated with major bleeding (Table 2). These complications did not influence the patient's ultimate outcome. There were no gastrointestinal bleeding events.

### Seroconversion of IgG-class PF4/heparin antibody

The seroconversion of IgG-class PF4/heparin antibody was confirmed in 27.5% (44/160) of patients undergoing TKA and in 14.0% (30/214) of those undergoing THA (Table 3). The incidence of seroconversion of IgG-class PF4/heparin antibodies was significantly higher in patients undergoing TKA compared to those undergoing THA. The seroconversion of IgG-class PF4/heparin antibodies varied with the different thromboembolic prophylaxis therapeutics. The conversion incidence of IgG-class PF4/heparin antibodies was significantly lower in patients receiving fondaparinux compared with those receiving UFH. Interestingly, seroconversion of IgG-class PF4/heparin antibodies was demonstrated in patients who did not receive UFH, LWMH or fondaparinux.

**Table 3:** Analysis of IgG-class anti-PF4/heparin antibody seroconversion rate

THA/TKA	drug	No. patients	No. seroconversion(%)	Total(%)
**THA**	UFH	60	17(28.3)	30(14.0)
	LMWH	64	4(6.3)*	
	Fondaparinux	49	4(8.2)**	
	others	41	5(12.2)	

**TKA**	UFH	44	17(38.6)	44(27.5)****
	LMWH	31	5(16.1)***	
	Fondaparinux	32	8(25.0)	
	others	53	14(26.4)	

Abbreviations: TKA; total knee arthroplasty, THA; total hip arthroplasty, UFH; unfractionated heparin, LMWH; low-molecular weight heparin, *p = 0.001 versus UFH, **p = 0.008 versus UFH, ***p = 0.035 versus UFH, ****p = 0.001 versus THA

### Platelet count

**Figure 1:**
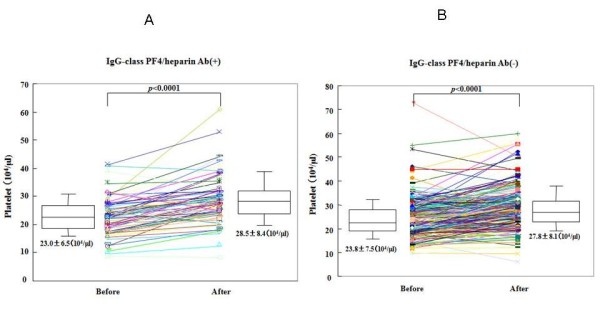
**Changes of platelet counts during the joint replacement treatment**. Platelet counts before operation and post-operation (post-operative day 7) in each patients with (A) or without (B) seroconvertion of IgG-class anti-PF4/heparin antibodies were shown.

We compared the platelet counts before the operation and at post-operative day 7 in the patients with or without seroconversion of IgG-class PF4/heparin antibodies. Platelet counts were elevated significantly at post-operative day 7 in the presence or absence of seroconversion of IgG-class PF4/heparin antibodies and none of these patients developed HIT (Figure 1).

### Risk factors for DVT

In comparisons between patients with or without DVT, several variables were selected through univariate analysis, based upon a p-value <0.1 (Table 4), and these selected parameters were then subjected to multivariate analysis. The parameters included were; age, gender, the type of operation (TKA/THA), and postoperative (7 days) seroconversion of IgG PF4/heparin antibody. Logistic regression multivariate analysis revealed older age (≧75 yr), female gender and seroconversion of the IgG anti-PF4/heparin antibody as significant independent risk factors (Table 5).

**Table 4:** Patients characteristics and symptomatic DVT

	DVD(-)		DVT(+)		
Characteristics	n = 318		n = 56		*p*
Age	68.2 ± 11.6	( 25-90 )	74.5 ± 7.8	(45-86)	<0.00001
Age≧75 yr	106		32		0.001
Age < 75 yr	212		24		
Gender(male/female)	60/258		5/51		0.070
BMI(Kg/m^2^)	24.7 ± 4.2	(13.7-41.4)	25.8 ± 3.8	(20.1-35.1)	0.088
BMI≧30	37		10		0.195
BMI < 30	281		46		
					
Risk factors	176(55.3%)		38(67.9%)		0.081
TKA/THA	125/193		35/21		0.001
OA/RA	273/46		48/7		0.697
Seroconvertion of IgG-class Anti-PF4/heparin Ab	54(17.0%)		20(35.7%)		0.001
Treatment					
UFH	89	(27.7%)	16	(28.6%)	0.890
LMWH	81	(25.5%)	14	(25.0%)	0.940
Fondaparinux	73	(23.0%)	8	(14.3%)	0.146
Others	37	(11.6%)	13	(23.2%)	
Aspirin	31	(9.7%)	12	(21.4%)	
Warfarin	5	(1.6%)	0		
Cilostazol	26	(8.2%)	11	(19.6%)	
No medication	39	(12.3%)	5	(8.9%)	0.475

Abbreviations: DVT; deep vein thrombosis; BMI; body mass index, TKA; total knee arthroplasty, THA; total hip arthroplasty, OA; osteoarthritis, RA; rheumatoid arthritis, UFH; unfractionated heparin, LMWH; low-molecular weight heparin

**Table 5:** Multivariate analysis for risk factors of symptomatic DVT

Variable	***p***	HR	95%CI
Age≧75 yr	0.010	2.396	(1.227-4.677)
Male Gender	0.041	0.353	(0.130-0.957)
Risk factors	0.760	1.114	(0.556-2.233)
TKA/THA	0.074	1.792	(0.944-3.401)
Seroconvertion of IgG-class HIT Ab	0.005	2.583	(1.338-4.984)

Abbreviations: DVT; deep vein thrombosis, TKA; total knee arthroplasty, THA; total hip arthroplasty, HR; Hazard Ratio, CI; Confidence Interval

Next, we determined whether seroconversion of the IgG-class HIT antibody contributed to the occurrence of DVT in the subgroups receiving the same antithrombotic prophylaxis agent (Table 6). Seroconversion of the IgG-class HIT antibody was significantly associated with the occurrence DVT in patients receiving UFH (*p *= 0.001, OR; 6.2). Meanwhile, seroconversion of the IgG-class HIT antibody was not associated with the occurrence of DVT in patients receiving LMWH or fondaparinux, as well as in patients who were not treated with these prophylaxes.

**Table 6:** Comparison of symptomatic DVT incidence between IgG-class anti-PF4/heparin Ab seroconverted and non-seroconverted patients

	Incidence of symptomatic DVT (%)			
Drug	seroconverted	non-seroconverted	Odds ratio (95%CI)	*p value*
				
UFH	11/34(32.4%)	5/70(7.1%)	6.217 (1.951-19.816)	0.001
				
LMWH	3/9(33.3%)	11/86(12.8%)	3.409 (0.743-15.642)	0.125
				
Fondaparinux	3/12(25.0%)	5/69(7.2%)	4.267 (0.868-20.972)	0.092
				
Others	3/19(15.8%)	15/75(20.0%)	0.750 (0.193-2.913)	0.481
				

Abbreviations: DVT; deep vein thrombosis, UFH; unfractionated heparin, LMWH; low-molecular weight heparin, 95%CI; confidence interval

## Discussion

In this report we describe our studies comparing the incidence of anti-PF4/heparin antibodies in patients receiving different thromboprophylaxis therapeutics after total joint arthroplasty (TKA and THA). Previous study has reported a significant difference in HIT between unfractionated heparin (UFH) and LMWH in postoperative orthopedic patients, suggesting that LMWH is safer than UFH in these patients [9,15]. Our hypothesis was that the frequencies of seroconversion of IgG-class anti-PF4/heparin would be reduced in patients receiving fondaparinux, and even in those receiving LMWHs, and we focused on the relationship between seroconversion of these antibodies and the occurrence of symptomatic DVT under different anti-thrombotic prophylaxis therapeutics. We found that seroconversion of the IgG-class anti-PF4/heparin antibody occurred in a significant number of patients and that it occurred more often in post-operative patients receiving UFH compared with those receiving fondaparinux or LMWH. Furthermore, we demonstrated that seroconversion of the IgG-class anti-PF4/heparin antibody could be an independent risk factor of symptomatic DVT in the absence of thrombocytopenia. Our data also demonstrated that older age (≧75 yr) is an independent risk factor of symptomatic DVT. Old age has already been demonstrated to be an important risk factor for VTE [3]. Our result seems to support these previous findings.

HIT is caused by heparin-dependent IgG-class antibodies against the PF4/heparin complex. These IgG immune-complexes bind to the platelet surface, resulting in platelet activation and aggregation [16]. Immune assays for PF4/heparin antibody generally detect antibodies of all classes Igs (IgG, A, and IgM) [12]. Accordingly, these immune assays can be expected to display much lower specificity, since only antibodies of the IgG class against PF4/heparin complex can activate platelets [13]. Therefore, in this prospective study, we used an IgG-specific EIA to improve the specificity for the anti-PF4/heparin antibody compared with the global assay. The diagnosis of HIT is based on clinical criteria, including thrombocytopenia, which is confirmed by *in vitro *demonstration of anti-PF4/heparin antibodies using functional and immunological methods [17]. Although functional assays are considered to represent the gold standard in the diagnosis of HIT, they are technically challenging and few laboratories have the ability to perform them.

Through the analysis of a large cohort of 374 patients, we observed that IgG-anti-PF4/heparin antibodies were frequently detected in patients receiving UFH, as well as those receiving LMWH or fondaparinux. Furthermore, seroconversion of the IgG-class anti-PF4/heparin Ab was an independent risk for symptomatic DVT in these patients. However, in the sub-analysis seroconversion of the IgG-class anti-PF4/heparin Ab was not an independent risk for symptomatic DVT in patients receiving LMWHs, fondaparinux or oral anti-coagulants. These observations suggest that seroconversion of the IgG-class anti-PF4/heparin Ab could contribute to the thromboembolic complications in patients receiving UFH, and not in those receiving LMWEs or fondaparinux.

The prevalence of HIT varies between agents and its range is 1-5% for UFH and 0-0.8% for LMWH [18]. Warkentin *et al. *reported the seroconversion incidence of anti-PF4/heparin Ab (fondaparinux 1.4% vs. enoxaparin 1.3%) and the incidence of HIT (fondaparinux 0.4% vs. enoxaparin 0.4%) in orthopedic surgery patient receiving fondaparinux and enoxoparin [9]. In our IgG-specific immune assay, the proportion that seroconverted was 32.6% in patients receiving UFH, 9.5% for LMWHs and 14.8% for fondaprinux. Although this study was limited because we did not use functional assays, these seroconversion rates of IgG-class anti-PF4/heparin Ab were shown to be relatively high with these anti-thromboembolic prophylactic therapeutics.

Non-drug factors, including type of surgery, are thought to play an important role in influencing the anti-PF4/heparin immune response. Ahamad et al. reported that anti-PF4/heparin antibody formation was higher in patients who had undergone total knee arthroplasty compared with total hip arthroplasty in clinical trial comparing UFH and LMWH [19]. More recently, Warkentin et al. demonstrated that anti-PF4/heparin antibody formation was more frequently observed after total knee arthroplasty compared with total hip arthroplasty [20]. In our study, in TKA, air tourniquest was used during operation in all patients. Hemostasis or local tissue ischemia of lower extremities related to the use of air tourniquet could partly contribute to the increased frequencies of IgG-class anti-PF4/heparin antibodies in patients receiving TKA.

It has been suggested that a syndrome resembling HIT occurs less frequently with fondaparinux [21]. This hypothesis is based on the concept that the IgG class anti-PF4/heparin antibodies are generated in fewer patients receiving fondaparinux [22]. Indeed, our study indicated that the seroconversion incidence of IgG-class anti-PF4/heparin antibody was lower in patients receiving fondaparinux compared with those receiving UFH. It has been demonstrated that fondaparinux does not activate platelets in the presence of sera obtained from patients with HIT, however UFH can activate platelets in functional platelet activation assays [23]. Also, previous studies have shown that sera obtained from patients with HIT containing anti-PF4/heparin antibodies fail to react against PF4 in the presence of fondaparinux [9]. These differences may contribute to our finding that seroconversion of the anti-PF4/heparin antibodies was not an independent risk factor for symptomatic DVT in patients receiving fondaparinux. Our results suggest that IgG-class anti-PF4/heparin antibodies generated in patients receiving fondaprinux were not able to activate platelets to induce thrombotic complications.

Our study has potential limitations. We surveyed symptomatic DVT in patients undergoing TKA or THA with different antithrombotic prophylaxis therapeutics. However, we attempted to maintain consistency between the patients, treatments and outcomes across the groups; i.e., all patients who underwent THA or TKA receiving standardized anticoagulant regimens, and had the same duration of follow-up. All thrombotic outcomes were confirmed with objective tests. Thus, we believe that our results provide valid estimates of the risk of symptomatic venous thromboembolism in patients who receive short-duration prophylaxis after THA or TKA. Another limitation of our study includes the performance rate of venous doppler US or computed tomography. Therefore, the incidence of DVT in the present study cannot be compared to that of previous studies. We surveyed the usage of anti-coagulants and anti-platelet agents, before and after total joint arthroplasty (TKA and THA), however, the natural products with anti-thrombotic properties had not been monitored.

In summary, symptomatic DVT developed in 15.0% of patients undergoing total joint arthroplasty (TKA and THA) with various thromboprophylaxis therapeutics. However, it was determined that the independent risk factors for DVT are elder-age (≧75 years) and seroconversion of the IgG-class PF4/heparin antibody. The use of UFH and TKA was associated with a higher incidence of IgG-class PF4/heparin antibodies compared with the use of LMWH or fondaparinux and THA. Furthermore, seroconversion of the IgG-class PF4/heparin antibody contributed to the occurrence of DVT more frequently in patients receiving UFH compared to those receiving LMWH or fondaparinux. These findings suggest that seroconversion of IgG-class PF4/heparin antibodies can be associated with DVT even in the absence of thrombocytopenia, in a subset of patients undergoing total joint arthroplasty (TKA and THA).

## Conclusion

Seroconvertion of IgG-class anti-PF4/heparin antibodies were occurred in a significant number of patients receiving total joint arthroplasty (TKA and THA) treatments and their frequencies may depend on anti-thrombotic prophylaxis agents and types of operation (TKA or THA). Furthermore, seroconvertion of IgG-class anti-PH4/heparin antibodies could be an independent risk factor for symptomatic DVT in these patients.

## Abbreviations

DVT: deep vein thrombosis; HIT: heparin-induced thrombocytopenia; LMWH: low molecular weight heparin; TKA: total knee arthroplasty; THA: total hip arthroplasty; PE: pulmonary embolism; UFH: unfractionated heparin; VTE: venous thromboembolism

## Competing interests

The authors declare that they have no competing interests.

## Authors' contributions

TT, KM, YM carried out the immunoassays. KM, SM, KO YJ HS participated in the design of the study and performed the statistical analysis. SM, SS TY conceived of the study, and participated in its design and coordination and helped to draft the manuscript. All authors read and approved the final manuscript.

## Pre-publication history

The pre-publication history for this paper can be accessed here:

http://www.biomedcentral.com/1471-2474/12/22/prepub
